# Hospital Festivals, Meetings, &c.

**Published:** 1894-03-10

**Authors:** 


### HOSPITAL FESTIVALS, MEETINGS, ETC.

### LONDON HOSPITAL.

A meeting of the Governors of the London Hospital was held on March 7th, with Mr. T. H. Buxton in the chair. The report for last year chronicled a daily average of 656 in-patients (10,599 in all) with an average of 24·04 days' residence, the total number of out-patients being 27,625. The receipts for 1893 amounted to £90,800, of which £34,000 were legacies; the expenditure for the year being £71,794, while £10,000 went to pay off a debt with which the year opened. The Chairman, in moving the adoption of the report, spoke of the management of the hospital as working always at full pressure, and alluded to the enormous amount of labour entailed by the out-patient department, which he characterized as an "excrescence" upon the primary work of the hospital. Mr. Hall, in reply, hoped that the chairman's speech did not mean that any curtailment of the work done by the out-patient department was projected; and in this he was supported by the Rev. Mr. Gedge, who advocated greater care in the distribution of out-patient's letters on the part of the governors.

The court then proceeded to elect Dr. Gilbart Smith Senior House Physician in the place of Dr. H. Jackson. Papers relating to the proposed memorial to the late Sir Andrew Clark were distributed, and the chairman announced that a public meeting for the consideration of the subject would soon be held.

### ROYAL WESTMINSTER OPHTHALMIC HOSPITAL.

The annual meeting of governors was held at this hospital on Friday, March 2nd, with Mr. Henry Power, M.B., in the chair. The report presented chronicled an increase in the number of patients during the last year, 9,634 having received treatment. The increase in the income of the hospital—£2,354, as against £2,096 in 1892—is accounted for by the larger amounts received in legacies. The Chairman referred to the urgent necessity for a new hospital to meet the needs of the increasing numbers of the patients. For this purpose £50,000 would be required, of which only a very small part has yet been collected; while the general income of the hospital has declined, the legacies received last year having alone prevented the existence of a serious deficit.

### SEAMEN'S HOSPITAL SOCIETY.

The annual court of governors of the Seamen's Hospital Society was held at the "Dreadnaught" Hospital, Greenwich, S.E., on February 28th, with Admiral Sir R. Vesey Hamilton in the chair. The report presented to the governors chronicled an excess of expenditure over income of more than £2,000, and as this is the third year in succession in which the receipts have been below the expenditure, the committee fear that they will have to curtail their sphere of usefulness if further help be not forthcoming. The number of patients received during the past year (including cases of accidents and severe illness among landsmen, which it would have been impossible to remove) was 17,749—a larger number than in any previous twelvemonth. The Chairman, in his introductory address, alluded to the need for an increase of the subscriptions in proportion to the work done by the institution. The adoption of the report was moved by Mr. P. A. Nairne, who referred to the question of admitting landsmen to the hospital; he thought that, although the institution was primarily intended for seamen, the committee should feel no hesitation in admitting urgent cases among landsmen, such, for instance, as that of the Anarchist Bourdin, brought a few days ago to the hospital in a dying condition. He also referred to the urgent necessity for a branch hospital at Tilbury, which, however, until sufficient funds were forthcoming, the committee did not feel themselves justified in beginning. The re-election of officers was moved by Mr. Day, and a vote of thanks to the chairman was passed. Among those present were Admiral Sir W. Graham, K.C.B., Admiral H. de Kantzow, Lieut.-Colonel W. E. Despard, Rear-Admiral E. S. Adeane, C.M.G., and G. Lidgett, Esq.

### PROVIDENT SURGICAL APPLIANCES SOCIETY.

The annual meeting of the above society was held on February 28th at the Great Eastern Hotel, with Mr. London in the chair. The receipts for the past year amounted to £2,345 19s. 11d., of which a balance of £47 remained to the credit of the society. The number of attendances of patients was 9,727, an increase of about 700 on the year before; and the number of instruments, &c., supplied to patients was 7,296, an increase of about 500. It will be seen from these figures that the society continues to make progress; its subscriptions also have somewhat increased during the year. It is, unfortunately, impossible to give more than this short summary of the position of the society as a report was not available, and the secretary, having been called away, could supply no information, while the proceedings at the meeting itself were of very short duration.

### THE NATIONAL ORTHOPÆDIC HOSPITAL.

The annual meeting was held on Wednesday last, Mr. Francis Hoare, in the chair. The annual report showed a slight increase upon the ordinary income, and detailed special contributions from City companies and others; 1,084 out-patients have received advice and attention, and 217 in-patients have been treated by the surgeons, the average stay of each patient being 98 days. The report recognised the services of the Ladies' Committee, and the professional staff, and in closing, pleaded for the removal of the rebuilding debt of £2,144, and a very much increased list of subscribers. The total income for the year was £1,710 0s. 4d., and the total expenditure £1,741 17s. 11d. Resolutions adopting the report and re-electing the committee and officers were passed unanimously.

### LONDON AND COUNTIES MEDICAL PROTECTION SOCIETY.

A meeting of the council of this society was held on February 27th, at 57, Harley Street, under the presidency of Dr. Heron, F.R.C.P. It was decided to remove the registered offices to 12, New Court, Lincoln's Inn, W.C., after the 31st inst. Messrs. Le Brasseur and Oakley, of the same address, were appointed solicitors to the society. A standing "legal committee," consisting of ten members, was appointed, to whom matters of a legal or defensive character will be submitted. A committee was also formed for the object of watching Parliamentary proceedings interesting to medical men. A vote approving the prosecution of the Indian oculists was unanimously passed. The question of changing the commencement of the society's year from May 1st to January 1st was discussed, and a resolution was passed directing the legal committee to revise and consider the existing articles of association, and report thereon to the meeting of council in April next. The list of candidates for membership was approved and all were elected.

